# A Comparative Review of Artificial Intelligence Applications in Small Molecule Versus Peptide Drug Discovery

**DOI:** 10.3390/ijms27073142

**Published:** 2026-03-30

**Authors:** Han Lin, Horst Vogel, Huawei Zhang

**Affiliations:** 1 Department of Biomedical Engineering, Southern University of Science and Technology, Shenzhen 518055, China; 2 Faculty of Pharmaceutical Sciences, Shenzhen University of Advanced Technology, Shenzhen 518107, China; 3 Center For AI-Driven Medical Research, Shenzhen Institutes of Advanced Technology, Chinese Academy of Sciences, Shenzhen 518055, China; 4 Institute of Chemical Sciences and Engineering, École Polytechnique Fédérale de Lausanne, 1015 Lausanne, Switzerland

**Keywords:** artificial intelligence, drug discovery, small molecules, peptides, machine learning, deep learning, generative AI, ADMET, virtual screening, de novo design

## Abstract

Traditional drug discovery processes are typically expensive, time-consuming, and have a very high failure rate. Artificial intelligence (AI) is currently reshaping this field in unprecedented ways, promising to significantly improve the efficiency and success rate of drug development. This article systematically compares and analyzes the application of AI for two major drug types: small molecule vs. peptide drugs. It explores their applications in several key stages of drug development, including virtual screening, lead compound optimization, de novo drug design, ADMET (absorption, distribution, metabolism, excretion, and toxicity) property prediction, and chemical synthesis planning. While both drug types benefit from AI-driven approaches, fundamental differences exist in molecular representation, data availability, key challenges, and model adaptability. For small molecule drugs, AI focuses on drug efficacy, synthetic feasibility, and accurate structure–activity relationship prediction. In contrast, for peptide drugs, AI faces more unique biological challenges, such as inherent flexibility, complex biological functions, stability, and immunogenicity. Finally, this article provides a forward-looking perspective on the future of AI-driven drug discovery, highlighting the immense potential of basic models, multimodal integrated systems, and autonomous discovery platforms, which will collectively drive the next wave of precision drug development.

## 1. Introduction

The traditional drug discovery process is lengthy and arduous due to the long development cycles and extremely high failure rates [1]. It is estimated that bringing a new drug from initial laboratory experiments to final market approval typically takes 10 to 15 years and costs up to $2.8 billion [2]. More importantly, 80% to 90% of drug candidates ultimately fail during clinical trials due to insufficient efficacy or safety concerns. This process heavily relies on the accumulated experience of medicinal chemists, repeated trial-and-error experiments, and a degree of serendipity, leading to low efficiency and difficulty in meeting the growing healthcare needs [3]. In recent years, artificial intelligence (AI), particularly its subfields of machine learning (ML) and deep learning (DL), has become a core driving force for paradigm shifts in this field. The fundamental advantage of AI lies in its ability to process and analyze massive, complex, and multidimensional biological and chemical data, thereby improving the speed and accuracy of drug development at every stage.

**Figure 1:**
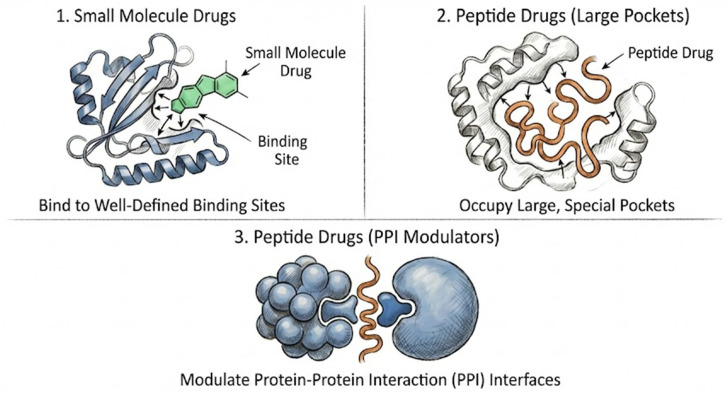
Different binding modes between small molecule drugs and peptide drugs. A small molecule drugs (green) usually binds to a well-defined sites in the target proteins (1), whereas a peptide drug (orange) can occupy a large special pocket (2) or modulate protein–protein interaction interfaces (3).

The development of AI-driven drug discovery presents unique opportunities and challenges for two main approaches: small molecule drugs and peptide drugs. As shown in Figure 1, small molecule drugs are the cornerstone of the pharmaceutical industry and typically function by interacting with specific “binding sites” on target proteins [4,5]. Peptide drugs, on the other hand, are used for emerging therapies positioned between small molecule drugs and biologics [6]. They have attracted considerable attention due to their high specificity, efficacy, and unique ability to target “undruggable” targets (e.g., targets with large binding pockets and those involved in protein–protein interactions (PPIs)) [7]. The fundamental differences in the physicochemical properties, biological behavior, and mechanisms of action of these two types of drugs necessitate the use of different strategies and models when applying artificial intelligence in their development.

This review aims to provide a comparative evaluation of AI applications in the core stages of drug discovery for both small molecule and peptide modalities, following the logical flow of the discovery process, and to forecast future developments in this rapidly evolving field.

## 2. Foundational Divergence: Molecular Language and Data Ecosystems

**Figure 2:**
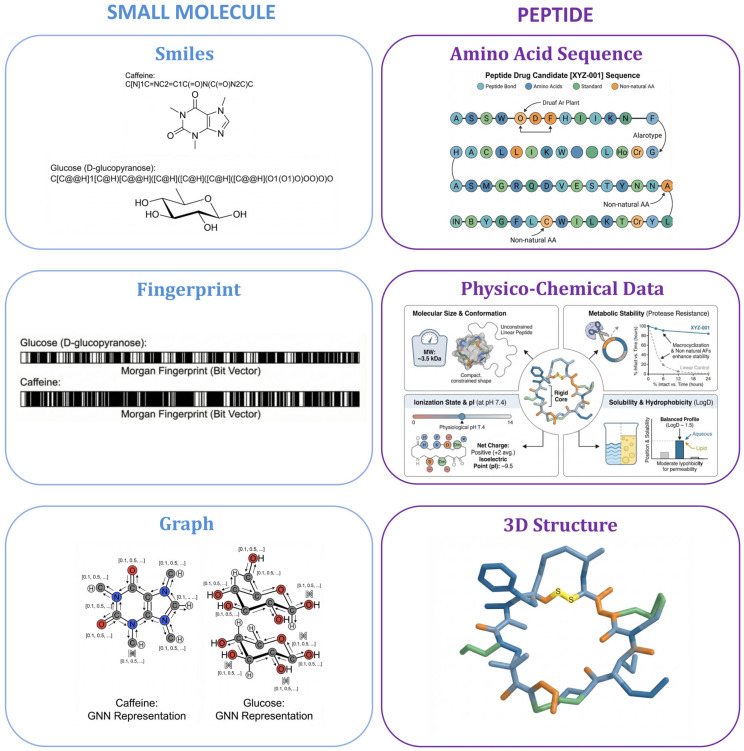
Different representations between small molecule drugs and peptide drugs. The left side and blue block: a small molecule drug using caffeine and glucose as an example; the right side and purple block: a peptide drug. A detailed description is given in the article.

The fundamental differences in AI strategies for small molecule and peptide drug discoveries stem primarily from two factors, the methods used for molecular representation (i.e., the “language” of the molecules) and the available structural data representing different properties, as shown in Figure 2, Table 1 and Table 2. The dominance of specific AI architectures in each modality is not accidental but reflects a co-evolutionary dynamical process where the inherent structural data of the molecules facilitate the development of customized computational tools.

For small molecules, three main representation strategies have been developed. The Simplified Molecular Input Line Entry System (SMILES [8]) provides a compact one-dimensional string format that is compatible with natural language processing [9] (NLP) models built on architectures such as transformers [10]. For example, in pre-trained large language models for chemistry (such as MolFormer [11] or ChemBERTa [12]), SMILES successfully enables the models to learn rich chemical properties, demonstrating excellent performance in downstream tasks such as solubility prediction and toxicity screening. However, SMILES also has significant limitations. First, it is a one-dimensional linear representation method, which inherently struggles to capture the three-dimensional conformational information of molecules. For example, when dealing with drug molecules with complex chiral centers or macrocyclic structures, the SMILES normalization process may lead to the loss of topological information. Second, subtle changes in SMILES strings (such as rearrangement of atom order) can lead to drastic fluctuations in the generated vector space, and this “brittleness” limits its robustness in generative models [13,14].

This non-unique representation syntax and the lack of three-dimensional information have prompted the development of more powerful alternatives, such as Self-Referencing Embedded Strings (SELFIES) [15], designed to consistently generate valid and legitimate molecular structures. Molecular fingerprints represent molecules as binary vectors, such as Extended Connectivity Fingerprints [16] (ECFPs), enabling fast similarity searches but at the cost of information loss. The current state-of-the-art approach is molecular graph representation [17,18], which treats atoms as nodes and chemical bonds as edges, such as graph convolutional neural networks [19] (GCNs) and graph attention networks [20] (GATs). This natural and information-rich format for small molecules serves as an ideal input for [17] GNNs, whose message-passing mechanism allows them to learn complex chemical patterns from the local atomic environment.

Molecular graphs, by directly modeling the connectivity between atoms, maintain the topological invariance of molecules. Regardless of the input order of the nodes, graph neural networks can extract consistent features through a message-passing mechanism, improving the model’s understanding of molecular structures [21]. Computer-aided drug design often needs to predict not only efficacy but also which part of the molecule is effective (e.g., by identifying pharmacophores). Graph neural networks with attention mechanisms (such as GATs) allow the model to assign different weights to different nodes during the encoding of the molecular graph. As demonstrated by the attentive FP model [22], graph-based encoding can visually show the specific atoms or functional groups that the model focuses on, thus providing interpretability for medicinal chemists.

Peptide representation, conversely, is rooted in biology. The most direct format is the one-dimensional amino acid sequence, a natural fit for NLP architectures [23]. There has been a rise of protein language models [24] (PLMs), such as ESM-2 [25], Ankh [26], and ProtTrans [27], and these models are large Transformer models pre-trained on massive databases of unlabeled protein sequences. By statistically learning evolutionary information between sequences, PLMs can generate powerful and informative embedding vectors that implicitly capture the structural and functional context of amino acid sequences without requiring explicit three-dimensional structures or sequence alignments—a key advantage given the relative scarcity of peptide-related data [28]. Systematic evaluations show that medium-sized PLMs typically achieve the best balance between performance and computational efficiency in downstream tasks. While three-dimensional structure remains functionally crucial, the immense conformational flexibility of peptides makes their structural representation a significant computational challenge.

Typically, PLMs perform well for peptide drugs composed of natural amino acids. For peptide drugs containing non-natural amino acids, several methods are employed, such as vocabulary expansion, atomic-level graph representation, and chemical descriptor embedding. Vocabulary expansion is the method closest to the traditional PLM logic. It treats each common non-natural amino acid as a new “token” and adds it to the model’s embedding layer for end-to-end training. A typical example is GPepT (https://huggingface.co/Playingyoyo/GPepT, accessed on 3 January 2026) [29], which creates a vocabulary containing over 17,000 non-canonical elements and uses the “Monomerizer” tool to decompose peptide chains into natural and non-natural amino acids. Because vocabulary expansion struggles to cover unseen structures, graph representation methods have been developed. These methods first represent atoms, then amino acids, and finally the peptide chain, processing the atomic graph of non-natural amino acids using GNNs to obtain the feature vector of the residue, such as PepMNet (https://github.com/danielgarzonotero/PepMNet, accessed on 3 January 2026) [30], which uses a hybrid deep learning architecture, capturing the chemical environment of side-chain atoms at the lower layer and modeling the amino acid sequence at the upper layer. For chemical string representations of peptide drugs, the approach is more similar to small molecule drugs, representing the entire peptide chain using SMILES or the more stable SELFIES strings, such as PeptideMTR (https://github.com/AaronFeller/PeptideMTR, accessed on 3 January 2026) [31], which directly encode peptides containing lipidation, PEGylation, and non-natural amino acids using a k-mer based SMILES tokenization strategy [31]. This approach demonstrates excellent performance for predicting aggregation propensity, tumor homing, membrane permeability, and antimicrobial activity.

These methodological differences stem directly from disparities in data quality and availability. The development in the small molecule field is based on mature, large-scale public benchmark datasets, such as DUDE-Z [32], which are carefully designed with attribute-matched decoy molecules to enable fair algorithm comparisons. Large databases like ChEMBL [33] and BindingDB [34] provide millions of curated quantitative structure–activity relationship [35] (QSAR) data (e.g., IC50, EC50, Kd, Ki), which are ideal for training supervised learning models, such as using EC50 data for a specific target to train a binary classification machine learning model as an initial screening tool to enrich small molecule drug targets that are likely to serve as novel agonists from a large-scale compound library [36]. In contrast, the peptide data landscape is highly fragmented, lacking “gold standard” benchmark datasets. Data are scattered across various specialized databases (e.g., PepBDB [37], SATPdb [38], DBAASP [39]) and primarily comprise raw sequence information, missing mostly functional data. This difference in data type dictates the learning paradigm: the abundance of labeled data allows small molecule AI to primarily develop within the supervised learning domain [40].

For example, the Chemprop (https://github.com/chemprop, accessed on 3 January 2026) model was used in a supervised learning task, training a binary classification model to determine whether a molecule has activity in inhibiting the growth of *Escherichia coli*. This led to the discovery of Halicin, a novel antibiotic capable of killing multiple drug-resistant bacteria [41].

On the other hand, the scarcity of labeled peptide data leads to the frequent use of transfer learning methods [42], relying on models pre-trained on broader protein sequence datasets. For example, researchers used the LLAMP model to collect a large dataset of 1.7 million peptide sequences to obtain a peptide sequence-optimized ESM-2 model. Using the encoded genomic information from various bacterial species to represent the target species, they finally fine-tuned the peptide sequence-optimized ESM-2 model and integrated the genomic information to create the LLAMP model, which can predict the MIC values of antimicrobial peptides based on peptide sequences and target bacterial species [43].

**Table 1:** Molecular representation methods for small molecules and peptides.

Representation	Modality	Description	Advantage	Disadvantage	Architectures
SMILES String	Small Molecule	Encodes molecular structure as a 1D ASCII string	Compact, easy to store, compatible with NLP models	Non-unique representation, fragile grammar, loses 3D information	RNN [44], Long Short-Term Memory (LSTM) [45], Transformer
Molecular Fingerprint	Small Molecule	Binary vector representing substructure presence	Fast computation, suitable for large-scale similarity search	Discrete, information loss (bit collision), weak similarity encoding ability	Classical ML (RF [46], SVM [47])
Molecular Graph (2D/3D)	Small Molecule	Graph structure with atoms as nodes and bonds as edges	Most natural representation, rich information, preserves topological/spatial structure	Complex generation, high computational cost for 3D conformation	GNN, CNN [48] (3D)
Amino Acid Sequence	Peptide	1D sequence composed of amino acid residues	Simple, intuitive, large data volume, easy to obtain	Ignores 3D structure and chemical modifications; similar sequences may have vastly different structures/functions	RNN, LSTM, Transformer
Sequence Embeddings (PLMs)	Peptide	Dense vectors extracted from large-scale protein language models	Captures context and evolutionary information, strong representation ability	Depends on the quality and coverage of pre-trained models	Transformer, MLP [49]
3D Structure	Peptide	3D coordinates of atoms or residues	Directly encodes function-related spatial information, high accuracy	Conformational flexibility leads to difficult representation, extremely high computational cost, data scarcity	GNN, SE(3)-Equivariant Networks [50]

**Table 2:** Key public datasets and benchmarks for small molecules and peptides.

Dataset	Modality	Description	Primary Use	Scale/Scope
DUDE-Z [32]	Small Molecule	Enhanced Useful Decoy Set, provides high-quality actives and property-matched decoys	Benchmark for structured virtual screening	Contains 102 targets, each with actives and 50× decoys
MUV [51]	Small Molecule	Maximum Unbiased Validation dataset, aims to minimize analog bias and artificial enrichment	Benchmark for ligand-based and structure-based virtual screening	Contains 17 targets, embedding actives within the chemical space of decoys
ChEMBL [33]	Small Molecule	Large public database containing drugs, targets, and their bioactivity data	Model training, QSAR, target discovery	Over 2 million compounds and 15 million activity data points
BindingDB [34]	Small Molecule	Focuses on binding affinity data for drug targets and small molecule interactions	Model training (affinity prediction)	Contains various affinity data such as Kd, Ki, and IC50
PepBDB [37,38]	Peptide	Curated database of peptide–protein complex structures from PDB	Structure-based peptide design and docking studies	Provides “clean” complex structures for computational research
SATPdb [38]	Peptide	Structurally annotated therapeutic peptide database, integrates 22 other databases	Browsing and searching for peptides with specific structures or functions	Over 19,000 unique therapeutic peptide sequences
THPdb [52]	Peptide	FDA-approved therapeutic peptide and protein database	Research on marketed drug properties	Contains sequence, mechanism of action, pharmacokinetics, etc.
DBAASP [39]	Peptide	Database of Antimicrobial Activity and Selectivity of Peptides	Research and design of antimicrobial peptides (AMPs)	Contains a large number of AMP sequences and their activity against different microorganisms

## 3. AI in Virtual Screening: A Tale of Speed Versus Flexibility

Computational methods are becoming increasingly important in modern drug discovery and have been widely applied in the pharmaceutical industry because computational tools play a crucial role at different stages of drug screening [53]. AI, as a computational tool, has played a significant role in virtual screening, which is one aspect of the drug discovery process. In the field of virtual screening, AI plays two distinct roles in two different forms of drug discovery, depending on the core limitations of each screening method. As shown in Table 2, for small molecule drugs, AI typically acts as an accelerator, aiming to address challenges related to scale and throughput. For peptide drugs, AI acts as an enabler, aiming to directly or indirectly address the fundamental physical problem of flexibility.

Traditional molecular docking software tools, such as AutoDock Vina (https://github.com/ccsb-scripps/AutoDock-Vina/releases, accessed on 3 January 2026) [54] or Glide (https://www.schrodinger.com/platform/products/glide/, accessed on 3 January 2026) [55], are computationally intensive, making screening billions of compound libraries a time-consuming task. AutoDock Vina is open-source, free, fast, and highly stable. Its empirical scoring function has been validated for many cases over a long time period and is suitable for initial large-scale virtual screening. However, its scoring function does not accurately capture complex polar interactions and cannot handle significant induced-fit effects. Glide, on the other hand, provides extremely high pose prediction accuracy through its Standard Precision (SP)/Extra Precision (XP) docking modes. It handles metal ions and water molecules exceptionally well, and it has a comprehensive graphical interface and ecosystem. However, it is commercial software, and its computational resource consumption is higher than Vina, making it unsuitable for screening billions of compounds without cluster support.

The main application of artificial intelligence lies in developing “surrogate models”—that is, training machine learning models to predict the results of classic docking scores at a significantly lower computational cost (Table 3). This facilitates a hierarchical workflow: fast AI models can quickly screen vast compound libraries, narrowing them down to a subset with high potential, which is then subjected to more rigorous traditional docking. This hybrid approach can increase screening speed by 10 to 100 times with virtually no loss of high-scoring compounds. Meanwhile, AI docking tools, by explicitly training on compound and pocket residue information, support the direct generation of reliable ligand–pocket poses, directly challenging traditional docking tools. Frameworks such as NeuralDock (https://github.com/Cromairis/Neuraldock/tree/main) (accessed on 3 January 2026) [56] and KarmaDock (https://github.com/schrojunzhang/KarmaDock, accessed on 3 January 2026) [57] have demonstrated that this principle can be used to screen nearly a billion molecules in less than a day.

NeuralDock [56] boasts extremely fast speed, implicitly handling conformational sampling and directly predicting binding energy, making it ideal for processing ultra-large compound libraries like ZINC. However, its performance is sometimes weaker in terms of the physical plausibility of predicted docking poses (e.g., bond lengths and angles), and it faces challenges in generalizing to novel, unseen binding pockets. KarmaDock [57], on the other hand, integrates three stages: pose generation, refinement, and affinity prediction. Utilizing E(n) equivariant graph neural networks (EGNNs), it maintains high speed while also considering the physical validity of the poses. However, its primary focus is on ligand flexibility, and its performance in simulating large-scale protein conformational changes is limited.

Concurrently, emerging co-folding tools like AlphaFold3 (https://github.com/google-deepmind/alphafold3, accessed on 3 January 2026) [58] and Boltz2 (https://github.com/jwohlwend/boltz, accessed on 3 January 2026) [59] are beginning to bypass traditional docking tools entirely, directly predicting protein–ligand complex structures end-to-end using sequence and SMILES inputs. AlphaFold3 possesses co-folding capabilities; beyond simple docking, it can simultaneously predict proteins, ligands, ions, and nucleic acids with high accuracy in blind docking, even requiring only the sequence. However, it has high inference costs and requires high-performance GPU support, and its performance in predicting ligand chirality and stereochemical constraints is not strong. Boltz-2 [59], on the other hand, is open-source and versatile. It not only predicts structures but also integrates an affinity prediction module, claiming to achieve FEP (free energy perturbation) accuracy while being fast. However, it still has high hardware memory requirements.

For peptide drugs, the challenge lies not in speed, but in feasibility. The large number of rotatable bonds in peptide chains creates a vast conformational space, which classical docking algorithms designed for semi-rigid small molecules cannot effectively sample. Therefore, the performance of these algorithms deteriorates sharply for peptides longer than five amino acids [60]. This is a fundamental physical sampling problem, not a computational throughput issue. The breakthrough with artificial intelligence lies in repurposing AI-driven protein structure prediction models, such as AlphaFold-Multimer (https://github.com/google-deepmind/alphafold, accessed on 3 January 2026) [61] and ESMFold (https://github.com/facebookresearch/esm, accessed on 3 January 2026) [62], for docking tasks. By treating the receptor and peptide as a single polypeptide chain connected by virtual flexible linkers, these tools transform the challenging “flexible docking” problem into a “structure prediction” or “co-folding” problem that the tools are adept at solving. This approach implicitly handles the flexibility of the peptide by predicting its binding conformation in the receptor environment.

AlphaFold-Multimer is specifically trained for predicting multimeric complexes. For short- to medium-sized peptides of up to 20 amino acids, it can often directly “fold” into binding conformations very close to the crystal structure. It also supports blind docking, autonomously searching the protein surface to find the most likely binding site and then fine-tuning the protein side chains based on peptide binding, thus simulating the induced fit effect to some extent. However, it does not directly support non-natural amino acids, and due to the randomness of sampling, it may get stuck in a single potential energy minimum, requiring a change in the degree of random number seed value to force sampling and capture more conformations. ESMFold [62], based on ESM-2, completely eliminates the MSA search step, inferring structure from a single sequence. Therefore, it boasts extreme speed, making it suitable for preliminary topological screening of massive peptide libraries. It also performs more robustly than MSA-based tools for orphan proteins or artificially designed peptides lacking homologous sequence information. However, it is less accurate than AlphaFold-Multimer in predicting fine-grained interactions (such as hydrogen bonds and salt bridges) and is extremely sensitive to interface selection.

Further refinement using methods such as molecular dynamics [63] (MD) simulations can be applied after docking to improve the accuracy of these predictions. MD simulations offer relative physical realism by simulating solvent (water, ion) effects and capturing the flexibility of peptide drug backbones. They are the only reliable method for handling special modifications such as cyclic peptides, non-natural amino acids, and lipopeptides. Combined with Molecular Mechanics/Poisson–Boltzmann Surface Area (MMPBSA) calculations or enhanced sampling techniques [64], they can quantitatively assess binding affinities. Representative tools include Gromacs (https://www.gromacs.org, accessed on 3 January 2026) [65] and Amber (https://ambermd.org/, accessed on 3 January 2026) [66,67]. However, MD simulations are extremely time-consuming, and without an initial conformation, it is almost impossible to observe binding within a limited time using equilibrium MD. MD simulations are also prone to getting stuck in local optima; if the initial docking pose is incorrect, the MD simulation may simply “move around aimlessly” in the wrong location.

## 4. The Role of AI in Optimizing Structure–Activity Relationship (SAR) Performance During Lead Optimization Tasks

Improving the SAR for small molecules is often a non-linear process. A change in a single atom (such as a methyl or fluorine atom) in a potential small molecule drug can lead to a hundredfold change in activity (the “magic methyl” effect). Optimizing the SAR of small molecule drugs means finding the optimal SAR features within a “steep” energy landscape of ligand–target interaction. The R group is a general symbol for various chemical substituents or functional groups attached to the small molecular backbone. Therefore, the impact of AI on such tasks is typically guided by predicting the effects of minute electronic and steric effects on binding energy, leading to precise substitution of R groups. For example, the generative model REINVENT (https://github.com/MolecularAI/REINVENT4, accessed on 3 January 2026) [68] developed by AstraZeneca in SAR optimization tasks finds the optimal substituent that simultaneously satisfies high activity and low toxicity (such as for hERG) while maintaining key interactions between the parent nucleus and the target (such as hydrogen bonds). REINVENT combines Stack-RNN with reinforcement learning (Policy Gradient) algorithms.

The LSTM networks are specially designed recurrent neural networks for learning long-range dependencies in sequential data. The function is achieved by learning the underlying statistical grammar of SMILES strings. The parameters of LSTM networks (including the trainable weight matrix and bias vector in its four gating units) parameterize the transition probabilities between characters and essentially learn the syntactic probabilities of SMILES strings, while the reinforcement learning [69] (RL) algorithm acts as a “navigator”. When the model generates a molecule with excellent SAR data (predicting a molecule of high activity), the RL algorithm calculates the gradient, the derivative of the reward function with respect to the model parameters. It tells the model in which direction the parameters should be fine-tuned to achieve a higher score (such as higher activity) and updates the LSTM parameters, increasing the probability of sampling a specific character from the predicted multinomial distribution over the SMILES vocabulary. Therefore, an AI model, like REINVENT, based on the LSTM process, revolutionizes traditional SAR research through three key mechanisms. 1. Autonomous exploration (active vs. passive): Unlike traditional models that merely predict the activity of user-provided molecules (passive), REINVENT autonomously generates new chemical structures using an LSTM-based generator (active). 2. Chemical spatial orientation navigation: This model uses reinforcement learning to act as a “navigator” in a multidimensional chemical space. It employs a reward-based feedback loop to move the generated molecular distribution towards “local” regions that satisfy multiple objectives, such as minimizing toxicity while optimizing lipophilicity. 3. Prioritized synthesis recommendations: This targeted convergence approach addresses the uncertainty inherent in traditional drug discovery—that the most promising next step is often unclear—by providing a computationally prioritized list of candidate compounds. This guides researchers to determine “what to synthesize next” with higher statistical confidence.

Another example is the Active Learning FEP+ (https://www.schrodinger.com/platform/products/fep/, accessed on 3 January 2026) [70] tool developed by Schrödinger, which optimizes the SAR of lead compounds by precisely quantifying the change in binding free energy [71] after introducing different substituents (Cl, F, OMe, etc.) at different positions on the molecular scaffold, such as on the benzene ring. The specific algorithm combines GCN and Active Learning algorithms. GCN transforms the molecular structure into graph embedding vectors, capturing the connections between atoms, while Active Learning introduces “uncertainty sampling.” This AI method does not aim to predict the activity of all molecules. Instead, it identifies the molecular structures that are least “understandable” by current SAR models and sends only these molecules for high-precision FEP physical calculations (i.e., rigorous MD simulations based on statistical mechanics). With a very small amount of calculated data, the AI quickly grasps the SAR rules of that specific framework (the model learns that, for example, “adding an electron-withdrawing group at a particular position will increase activity”), thereby rapidly improving the activity for example from micromolar to nanomolar concentration range. However, Active Learning FEP+ has significant limitations. FEP requires molecules with similar core scaffolds. If AL suggests a completely different molecular structure, FEP may fail to establish a reasonable perturbation path, leading to calculation failure. Furthermore, the prediction error of the surrogate model can cause AL to get stuck in a local optimum. If the initial FEP results have systematic biases (e.g., inaccurate receptor parameterization), the model will continuously reinforce these erroneous predictions. Running AL-FEP requires frequent switching between GPU tasks (FEP sampling) and CPU tasks (model training and sampling). The underlying principle of FEP is still classical force fields. If the initial conformation of the protein pocket is incorrect, or if there are errors in the arrangement of key water molecules, the results calculated by AI will also be incorrect [72,73].

For peptide drug SAR optimization, the task is akin to pinpointing conformational SAR features within a vast sequence space. Because a peptide’s SAR is highly dependent on sequence-induced secondary structure changes (such as helix stability), changing a single amino acid not only affects side-chain interactions with the target but can also cause conformational changes of the peptide, which may lead to suppression of binding to the target. The role of AI here is typically to predict the combined impact of point mutations on overall folding stability and target binding interfaces. For example, Meta AI’s protein large language model ESM-2 can be used to determine which amino acid in the peptide sequence can be replaced by maintaining or even improving the peptide’s biophysical stability, ultimately optimizing the SAR. The specific algorithm of the ESM-2 model combines Transformer (Masked Language Modeling [74], MLM) and self-attention algorithms. Using the Transformer’s self-attention mechanism, it captures direct interactions between long-range distant residues in the sequence. By calculating the masked reconstruction probability (the model’s ability to predict a hidden amino acid based on its surrounding sequence context), the algorithm provides a score reflecting the “evolutionary rationality” of the mutation. Mathematically, this is expressed as the log-likelihood score of a mutation. A high score indicates “evolutionary rationality,” meaning the mutation conforms to the conserved patterns seen in millions of years of protein evolution. Thus, the algorithm of this approach predicts whether a certain mutation in the peptide’s amino acid sequence improves or disrupts peptide binding to the target protein.

However, applying ESM-2 to peptide drug optimization has certain limitations. Because ESM-2 is pre-trained using only natural amino acid sequences, its vocabulary is strictly limited to these 20 standard amino acids. Therefore, ESM-2 cannot accurately characterize or predict peptide drugs containing non-natural amino acids. Furthermore, ESM-2 struggles with extremely short peptides [25,75]. In SAR optimization, the properties of very short peptides (e.g., peptides with fewer than five amino acid residues) are significantly affected by terminal group charge and solvent effects. Since the ESM-2 training set primarily consists of long-chain proteins, its performance may be inferior to traditional QSAR models based on physical descriptors when dealing with these short peptides governed by physicochemical rules rather than evolutionary principles [25,76].

Another example is the Uni-Mol (https://github.com/deepmodeling/Uni-Mol, accessed on 3 January 2026) [77] model, which predicts changes in the peptide binding to the target protein after introducing non-natural amino acids (such as phenylalanine derivatives with large side chains) in the peptide sequence. While traditional deep learning is limited by coordinate system orientation, where the same molecule can yield different numerical representations depending on its arbitrary orientation in 3D space, the SE(3) equivariant Transformer can extract rotationally and translationally invariant geometric features. When non-natural amino acids are introduced, the spatial volume and shape of the side chains change. Based on SE(3) equivariant network algorithms, Uni-Mol can accurately capture the changes in local environment fingerprints caused by these changes in the volume of the non-natural amino acid side chains. This allows it to evaluate whether the modifications fill the cavity of the target binding pocket or whether it causes unreasonable atomic clashes. This helps to address the limitations of traditional sequence models, which cannot handle non-natural amino acids (because they are not present in the training data), while also accurately predicting the binding affinity after modification.

Uni-Mol critically depends on the quality of the input 3D conformation. Furthermore, the single Uni-Mol encoder primarily focuses on the structure of the peptide itself. In SAR optimization, if activity changes are caused by induced fit of receptor side chains, a Uni-Mol drug–target interaction (DTI) variant is needed to effectively capture the interactions at the interface. While Uni-Mol performs excellently for short peptides containing 5–15 amino acid residues, the computational cost and memory consumption increase significantly when processing long peptides or mini-proteins exceeding 50 amino acid residues. Unlike ESM-2, which derives evolutionary constraints and functional semantics from massive sequence databases, Uni-Mol aims to learn physicochemical characterizations directly from three-dimensional spatial coordinates. Therefore, Uni-Mol prioritizes geometric and atomic features but may lack the explicit evolutionary context (often referred to as “biological intuition”) inherent in language models. It knows whether a residue is stable in physical space, but it does not know whether that residue is evolutionarily conserved. For naturally derived peptide lead compounds, ignoring evolutionary constraints may lead to the design of sequences that are physically feasible but biologically ineffective.

**Figure 3:**
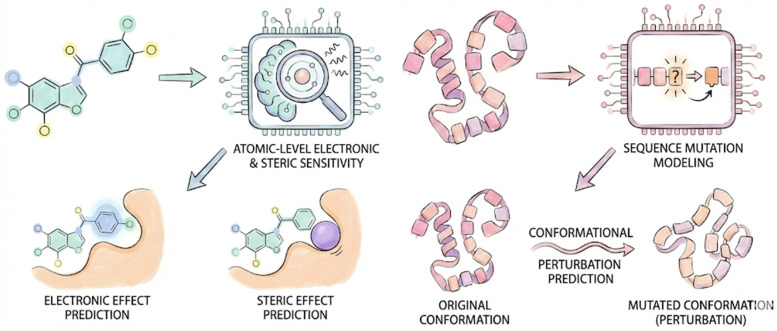
AI demonstrates different advantages for small molecule drugs and peptide drugs. (**Left**): Schematic representation of binding a small molecule drug to a target protein. AI is used to calculate and predict changes in atomic-level electronic and steric effects in small molecule drugs, in order to achieve lead optimization in SAR studies. (**Right**): Schematic representation of binding a peptide drug to a target protein. AI is used to calculate and predict the impact of amino acid sequence mutations on the spatial conformation of peptide drugs, thereby achieving lead optimization in terms of SAR.

AI exhibits fundamental differences in its approach for building and utilizing the SAR in drug lead optimization, as depicted in Figure 3. In the optimization of structure–activity relationships of small molecules to the target protein, the core task is to calculate and predict the sensitivity to atomic-level electronic and spatial effects. In contrast, in the optimization of structure–activity relationships of peptide drugs, the core task is to calculate and predict the spatial conformational perturbations caused by sequence mutations of the peptide drug to its binding to the target protein.

## 5. De Novo Design: Contrasting Philosophies of Property-First vs. Structure-First

**Figure 4:**
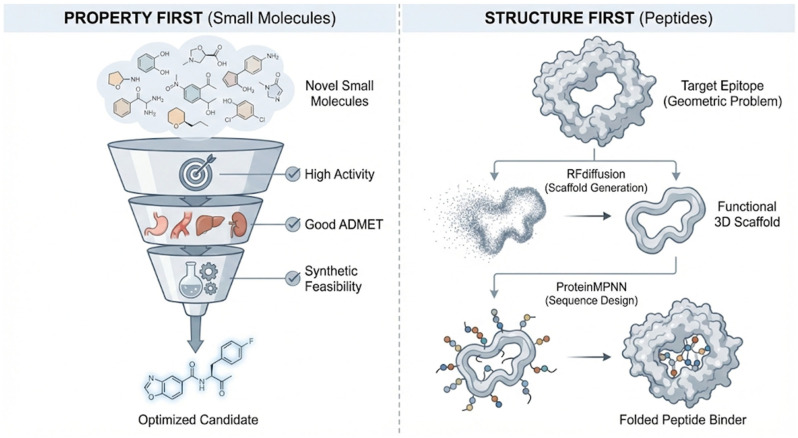
Difference in the generation of small molecule vs. peptide drugs. (**Left**): For small molecule drug design, “property-first” counts. (**Right**): For peptide drug design, “structure-first” counts.

Although newly designed generative AI frameworks have led to powerful models like diffusion models, which are a class of generative frameworks that create novel data by learning to reverse a multi-step noising process, the different performances of these tools when applied to the workflow of small molecule and peptide drug design reveal profound conceptual differences, as shown in Figure 4 and Table 4.

The “property-first” concept dominates the development of small molecule drugs. Its main goal is to create novel small molecules that satisfy a range of abstract and often difficult-to-reconcile properties, including high target activity, favorable absorption into the systemic circulation, distribution to target tissues, metabolism, excretion, and toxicity (ADMET) characteristics, and, crucially, synthetic feasibility. This is treated as a multi-objective optimization (MOO) problem [78], with models like MolProphet [79] utilizing methods such as reinforcement learning to explore the high-dimensional property space and find Pareto optimal solutions [80]—a set of candidate molecules representing the best possible trade-offs where no single property can be improved without sacrificing another. This approach acknowledges the inherent conflicts between drug potency, safety, and manufacturability, providing a diverse pool of leads for further optimization. However, small molecule drugs designed using this approach often face another major challenge: the “synthetic feasibility gap”—the difference between computationally “ideal” molecules and those that can actually be synthesized in the laboratory. Simple metrics like SAscore [81] often fail to accurately predict actual synthetic tractability and complexity. Therefore, advanced models like SynFormer [82] have been developed to address this problem, not only for generating molecules but also designing feasible synthesis routes, thus ensuring that synthetic feasibility is a core component of the design process, rather than an afterthought.

Peptide drug design follows a “structure-first” concept, based on the biological principle that “structure determines function”. Its goal is not to optimize abstract property scores but to solve a specific geometric problem: designing a physical three-dimensional molecular structure that can bind to a specific epitope on a target protein. The breakthrough tool in this field is RFdiffusion (https://github.com/RosettaCommons/RFdiffusion, accessed on 3 January 2026) [83], a diffusion model that generates novel and physically plausible protein or peptide scaffolds complementary to the target surface shape. Compared to methods for designing small molecule drugs, the design process is reversed. First, a functional three-dimensional scaffold is generated. Then, sequence design tools such as ProteinMPNN (https://github.com/dauparas/ProteinMPNN, accessed on 3 January 2026) [84] are used to find amino acid sequences that can stably fold into the predetermined scaffold. This paradigm has been successfully extended to designing cyclic peptides using RFpeptides [85], shifting the focus from optimizing numerical scores to creating functional physical entities, a method more aligned with biological intuition and fundamental principles.

The reasons for the differences in the design principles of small molecule drugs and peptide drugs can be analyzed from two perspectives: Firstly, from the fundamental differences in the importance of parameters (the intrinsic reason). Small molecules have relatively simple spatial structures, and the hurdle of functioning as a drug is extremely high, concerning the need to meet stringent requirements for membrane permeability, metabolic stability, and safety [86]. Peptides, on the other hand, have great structural flexibility. Without a stable secondary or tertiary structure, they cannot form an effective binding interface (especially for PPI targets), so their structure is a prerequisite for achieving sufficient affinity [87]. Secondly, the research hotspots and limitations of tools in the AI-driven drug design community (human/technical reasons) are discussed. With the development of AlphaFold2 (https://github.com/google-deepmind/alphafold, accessed on 3 January 2026) [88] and RosettaFold (https://github.com/RosettaCommons/RoseTTAFold, accessed on 3 January 2026) [89], peptide/protein structure prediction has become feasible and highly reliable, and researchers tend to focus on areas supported by advanced tools. Furthermore, there is currently a lack of high-throughput, high-quality peptide ADMET databases [90]. In contrast, the evaluation of small molecule drug-likeness has decades of accumulated experience and mature models.

## 6. From Bits to Beakers: AI’s Role in Synthesis Planning

**Figure 5:**
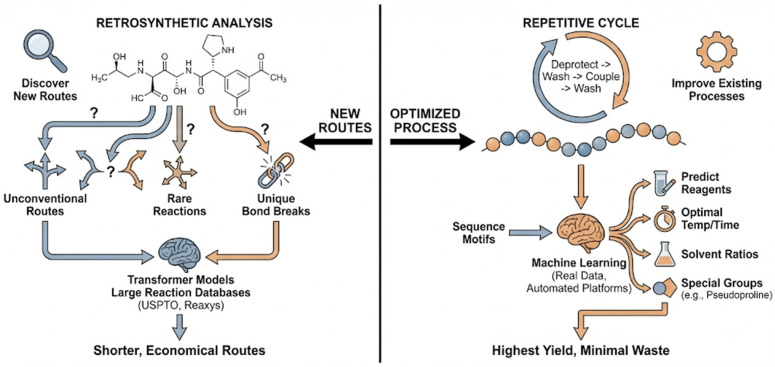
AI plays different roles in the synthesis design of two types of drugs. (**Left**): In the field of small molecule drugs, AI plays the role of an explorer, focusing on discovering new synthesis routes. (**Right**): For peptide drug synthesis, artificial AI plays the role of an optimizer, improving existing process workflows.

The application of AI tools for synthetic route planning clearly reflects the inherent differences in chemical characteristics between two different types of drug synthesis methods, as shown in Figure 5.

In the diverse and non-standardized field of organic small molecule chemistry, AI acts as an explorer, focusing on discovering new synthetic routes. These characteristics contribute to the different synthetic routes. First, the diversity of chemical structures: the small molecule organic chemical space offers immense possibilities, with each target molecule having a distinctly different backbone, functional groups, and stereochemical structure [91]. Second, there is no universal method for chemical synthesis: no single procedure can be applied to all molecules. For example, the method for synthesizing aspirin is completely different from the method for synthesizing paclitaxel [92]. A complex drug molecule may require 10, 20, or even more steps to synthesize, with dozens of possible reaction choices at each step. Therefore, in the field of retrosynthetic analysis, artificial intelligence primarily addresses the question of “how to synthesize?” Learning reaction routes from millions of scientific papers, and just like how AlphaGo [93] calculates chess moves, it can work backward from the target molecule to plan synthetic routes that human chemists might not consider, breaking free from traditional thinking, discovering rare reactions, or utilizing unique bond-breaking methods, thereby finding shorter, more economical, or easier-to-implement synthetic routes. AI models, particularly those based on the Transformer architecture, learn the fundamental principles of organic chemistry in small molecule design by training on large reaction databases (such as USPTO [94] and Reaxys [95]), ultimately enabling retrosynthetic analysis. Platforms like ASKCOS (https://askcos.mit.edu/, accessed on 3 January 2026) [96] and IBM RXN (https://rxn.app.accelerate.science/rxn/, accessed on 3 January 2026) [97] automate this process, providing chemists with multiple feasible synthetic routes for evaluation and selection.

In the standardized and process-driven field of peptide chemical synthesis, AI plays the role of an optimizer and is used to improve existing processes. For most peptide syntheses (especially solid-phase peptide synthesis, SPPS [98]), the core chemical reactions are repetitive and relatively simple: deprotection → washing → coupling → washing. Furthermore, the linear amino acid sequence itself dictates the synthesis order (usually from the C-terminus to the N-terminus), so the synthesis pathway is generally known. However, as the chain length increases, problems such as aggregation, folding, low coupling efficiency, and racemization may occur during synthesis, leading to reduced purity or synthesis failure. Artificial intelligence can address the question of “how to do it better.” It can directly predict suitable coupling reagents, required reaction temperatures, optimal solvent ratios, and reaction times. By training on large amounts of real-world synthesis data from automated platforms, machine learning models can learn the subtle relationships between sequence motifs and synthesis efficiency. The AI model acts like a process control engineer, flagging problematic sequences and providing optimized parameters (e.g., alternative reagents, extended reaction times), thus addressing problems before they even arise. For example, by analyzing sequence characteristics, AI can predict in advance which fragments are prone to aggregation and suggest introducing special protecting groups or “breaking” structures (e.g., pseudoproline [99]) at specific steps. Then, through iterative learning and optimization on the model using a small amount of experimental data, the optimal process conditions can be quickly found, resulting in the highest yield and minimal waste [100].

## 7. Predicting Biological Function: The Distinct ADMET Challenges

Predicting the ADMET characteristics of drugs reveals another fundamental difference between the two fields, as shown in Table 5. For small molecule drugs, ADMET prediction is primarily a chemical problem, focusing on the intrinsic physicochemical properties of the compound itself. For peptide drugs, however, it is a more complex biological problem, with characteristics depending on the interaction of the molecule with dynamic biological systems.

Because corresponding large public datasets (e.g., Tox21 [101], ClinTox [102], SIDER [103]) and established in vitro assay methods exist, predictions of the ADMET of small molecule drugs often yield good results. These data and methods can be used to train robust QSAR models. The focus of these predictions is on classic pharmacokinetic and toxicological parameters, such as metabolism by hepatic cytochrome P450 (CYP) enzymes [104], blood–brain barrier (BBB) permeability [105], and organ-specific toxicity, such as cardiotoxicity caused by inhibition of the hERG potassium channel [106].

In contrast, predicting the ADMET properties of peptide drugs faces challenges such as data scarcity and the complexity of potential biological interactions. The main metabolic challenge for peptide drugs is not protection against CYP enzymes [107] but rather rapid degradation by proteases; therefore, predicting their protease cleavage sites is crucial for designing stable peptide drugs. Due to their large molecular weight and high polarity, peptide drugs generally have poor cell membrane permeability, making the prediction of transmembrane properties a cutting-edge challenge. The main toxicity issue with peptide drugs is immunogenicity. Addressing immunogenicity involves predicting whether peptide fragments will bind to the highly polymorphic major histocompatibility complex [108] (MHC) molecules in individuals and trigger T-cell-mediated immune responses. This is a complex problem in the field of immune-informatics, and its difficulty is several orders of magnitude higher than predicting the single-target toxicity of small molecule drugs.

This duality is perfectly exemplified in the concept of “drug similarity”. For small molecule drugs, the definition is based on a priori physicochemical rules, such as Lipinski’s rule of five [109]. For peptide drugs, the criteria are more biologically based: proteolytic stability, tissue permeability, and low immunogenicity. These two sets of rules are almost entirely non-overlapping, thus requiring the development of completely different AI models and optimization objectives for the two drug types.

**Table 5:** Comparison of ADMET prediction challenges and AI methods for small molecules and peptides.

ADMET Prediction	Small Molecules	Peptides
Absorption/ Permeability	Description: Predict oral absorption-related properties, like Caco-2 permeability [110], blood–brain barrier permeability. Datasets: Caco-2 [110], BBBP [111], etc. AI Methods: QSAR models (GNN, RF, SVM).	Description: Generally poor permeability; predicting passive diffusion and active transport is a major challenge. Datasets: Data is scarce, mostly internal or from small literature sets. AI Methods: Sequence- and structure-based predictive models, still in exploratory stages.
Metabolism/ Stability	Description: Predict whether metabolized or inhibited by CYP450 enzymes. Datasets: Large public datasets on CYP inhibition/substrates [51,102]. AI Methods: Classification/regression models for CYP subtype specificity.	Description: The main challenge is rapid degradation by proteases, leading to a short half-life. Datasets: Protease cleavage site databases, but data is limited. AI Methods: Predict protease cleavage sites to guide sequence modification for enhanced stability.
Distribution	Description: Predict plasma protein binding [112] (PPB) and volume of distribution (Vd). Datasets: Public PPB datasets [113,114]. AI Methods: QSAR regression models.	Description: Distribution is limited by size and stability; targeted delivery is key. Datasets: Extremely scarce. AI Methods: No mature, general predictive models yet.
Toxicity (Specific)	Description: Predict hERG potassium channel inhibition (cardiotoxicity), a major cause of drug withdrawal. Datasets: Large public hERG activity datasets [115,116]. AI Methods: High-precision classification/regression QSAR models.	Description: Generally not a primary toxicity concern for peptides.
Toxicity (Systemic)	Description: Not a major consideration for most small molecules (except haptens).	Description: The most critical and complex safety risk is immunogenicity; prediction of T-cell/B-cell epitopes. Datasets: IEDB [117] and other immune epitope databases, but data is complex and HLA genotype-dependent. AI Methods: Prediction of peptide-MHC binding, a frontier in immunoinformatics.

## 8. Conclusions and Future Perspective

This review systematically compared the applications of AI in small molecule and peptide drug discovery, revealing that AI is not a one-size-fits-all solution. Its successful application largely depends on adapting to the unique chemical and biological characteristics of the drug type. Fundamental differences in molecular representation and data systems within the AI field have driven the co-development of different model architectures—GNNs for small molecule drugs and pre-trained language models inspired by natural language processing for peptide drugs. These differences permeate the entire drug discovery process, influencing the specific roles of AI in virtual screening (increasing throughput vs. enhancing flexibility), de novo design (optimizing abstract properties vs. constructing physical structures), ADMET prediction (addressing chemical vs. biological problems), and synthesis route planning (exploring synthetic routes vs. optimizing synthesis processes).

Despite significant progress in the application of AI in drug discovery, three major challenges remain. First, in this data-driven era, the lack of high-quality, large-scale, and standardized data remains a major bottleneck, a problem particularly pronounced in the peptide field, especially in predicting complex endpoints such as PPI ADMET properties. Second, the “black box” nature of many deep learning models poses a significant obstacle to trusting the application of the models themselves, highlighting the urgent need for advancements in explainable artificial intelligence [118] (XAI) technologies. Finally, the ultimate measure of success in drug discovery is clinical translation. While many drugs have shown promising preclinical results, the number of AI-designed drugs that successfully pass clinical trials and ultimately reach the market remains very small, highlighting the significant gap between computational predictions and actual therapeutic efficacy [119].

Looking ahead, several key trends are shaping the future of artificial intelligence in drug discovery. The convergence of AI design, automated synthesis, and high-throughput screening is giving rise to autonomous discovery platforms—closed-loop systems capable of automated, iterative design, build, test, and learn cycles. Furthermore, the field of drug discovery is shifting towards basic models, and large models pre-trained on massive multimodal datasets are used to learn the general principles of chemistry and biology. These models can be fine-tuned for various specific downstream tasks, providing more powerful and context-aware solutions. Finally, the field is moving towards multimodal integration, developing models capable of simultaneously processing and learning from multiple data types (e.g., text, sequences, 2D graphs, and 3D structures). Early examples, such as combining chemical and protein language models to predict interactions, foreshadow a future where AI will enable a more comprehensive and accurate understanding of complex biological systems. As AI technologies mature, they are poised to usher in a new era of faster, more cost-effective, and more precise drug development.

## Figures and Tables

**Table 3:** Representative AI-driven molecular docking tools.

Modality	Representative Tool/Method	Core Problem Solved	AI Role
Small Molecule	AI Surrogate Model (Lean Docking), AlphaFold-Ligand	Throughput: Accelerating screening of large databases	Fast filter/ end-to-end predictor
Peptide	AlphaFold-Multimer, ESMFold	Flexibility: Handling the vast conformational space of peptide chains	Structure predictor (transforms docking into a folding problem)

**Table 4:** Comparison of representative AI-driven de novo design tools.

Modality	Representative Tool/Method	Core Design Philosophy	AI Roles
Small Molecule	MOO frameworks (e.g., MolProphet), SynFormer	Property-First: Balance multiple abstract objectives (activity, ADMET, SA)	Multi-property optimizer/ Synthesis pathway planner
Peptide	RFdiffusion, RFpeptides	Structure-First: Design a functional 3D backbone that binds a target	Structural architect/Geometric problem solver

## Data Availability

No new data were created or analyzed in this study. Data sharing is not applicable to this article.

## Abbreviations

AI	Artificial Intelligence
AL	Active Learning
ML	Machine Learning
DL	Deep Learning
ADMET	Absorption, Distribution, Metabolism, Excretion, and Toxicity
PPI	Protein–Protein Interaction
SMILES	Simplified Molecular Input Line Entry System
GNN	Graph Neural Network
PLM	Protein Language Model
SA	Synthetic Accessibility
MOO	Multi-Objective Optimization
SPPS	Solid-Phase Peptide Synthesis
HTS	High-Throughput Screening
VS	Virtual Screening
SAR	Structure–Activity Relationship
QSAR	Quantitative Structure–Activity Relationship
RL	Reinforcement Learning
LLM	Large Language Model
MD	Molecular Dynamics
FEP	Free Energy Perturbation
XAI	Explainable Artificial Intelligence
